# Suicide risk in persons with polycystic ovarian syndrome: a systematic review

**DOI:** 10.1186/s12991-025-00574-w

**Published:** 2025-06-02

**Authors:** Sabrina Wong, Gia Han Le, Heidi Ka Ying Lo, Bing Cao, Poh Khuen Lim, Taeho Greg Rhee, Roger Ho, Hernan F. Guillen-Burgos, Kayla M. Teopiz, Lee Phan, Joshua D. Rosenblat, Melanie Zhang, Roger S. McIntyre

**Affiliations:** 1 Brain and Cognition Discovery Foundation, 77 Bloor Street West, Suite 617, Toronto, ON M5S 1M2 Canada; 2 Mood Disorder Psychopharmacology Unit, University Health Network, Toronto, Canada; 3 Department of Pharmacology & Toxicology, University of Toronto, Toronto, Canada; 4 Institute of Medical Science, University of Toronto, Toronto, Canada; 5 Department of Psychiatry, University of Hong Kong, Hong Kong, China; 6 Department of Psychiatry, University of Toronto, Toronto, Canada; 7 Key Laboratory of Cognition and Personality, Faculty of Psychology, Ministry of Education, Southwest University, Chongqing, 400715 People’s Republic of China; 8 Department of Psychological Medicine, Yong Loo Lin School of Medicine, National University of Singapore, Singapore, Singapore; 9 Institute for Health Innovation and Technology (iHealthtech), National University of Singapore, Singapore, Singapore; 10 Division of Life Science (LIFS), Faculty of Science, Hong Kong University of Science and Technology, Hong Kong, China; 11 Department of Psychiatry, Yale School of Medicine, New Haven, CT USA; 12 Department of Public Health Sciences, University of Connecticut School of Medicine, Farmington, CT USA; 13 Department of Psychiatry and Mental Health, Pontificia Universidad Javeriana, Hospital Universitario San Ignacio, Bogota, DC Colombia; 14 Center for Clinical and Translational Research, Universidad Simon Bolivar, Barranquilla, Colombia; 15 Center for Clinical and Translational Research, Universidad El Bosque, Bogota, DC Colombia

**Keywords:** Polycystic ovarian syndrome, Suicide, Depression, MDD, TRD, PCOS, Insulin resistance, Metabolic syndrome

## Abstract

**Background:**

Polycystic ovarian syndrome (PCOS) is a common and increasingly prevalent reproductive and metabolic endocrine disorder that is characterized by metabolic alterations, hyperandrogenism, menstrual irregularities as well as an increased risk of depression. Available evidence suggests PCOS may also be associated with disparate aspects of suicidality. Herein, we sought to determine the prevalence of suicidal ideation, suicidal behaviours and completed suicide in the PCOS population.

**Methods:**

We systematically searched PubMed, Ovid and Scopus databases from inception to January 7, 2024. A manual search was conducted on Google Scholar. Two reviewers independently screened the retrieved studies against the eligibility criteria (S.W. and G.H.L.). Human studies investigating suicide outcomes in women of reproductive age with a confirmed diagnosis of PCOS were included.

**Results:**

Eleven studies meeting our eligibility criteria were included. Although results were mixed, available evidence suggests that persons with PCOS are at an increased risk of suicidal ideation, self-harm and suicide attempts and are also differentially affected by psychiatric comorbidities (e.g., depressive disorders). Notwithstanding, suicide risk was not fully accounted for by the presence of mental illness, which suggests that PCOS may also be contributory.

**Conclusion:**

PCOS is associated with an increased risk of suicidal ideation and behaviour and associated psychiatric comorbidities. Persons with PCOS should be routinely evaluated for the presence of clinically significant suicidality. Whether increased suicidality in PCOS populations is a direct effect of the disease state and/or is largely moderated by psychiatric comorbidity is a future research vista.

**Supplementary Information:**

The online version contains supplementary material available at 10.1186/s12991-025-00574-w.

## Introduction

Polycystic ovarian syndrome (PCOS) is a female reproductive and metabolic endocrine disorder characterized by hyperandrogenism, ovulatory dysfunction and metabolic disruption (e.g., obesity, insulin resistance) [37]. Other common PCOS symptoms include acne vulgaris, increased body mass index, hirsutism, and alopecia [10]. PCOS affects approximately 8–13% of women of reproductive age, which may be an underestimate insofar as 70% of cases are undiagnosed [43]. The pathoetiology of PCOS is not fully ascertained but involves alterations in homeostatic adjustments of steroidogenesis, folliculogenesis, neuroendocrine function, metabolism, insulin signaling and sensitivity and inflammation [38]. Currently, there are no FDA-approved treatments for PCOS and/or interventions known to be disease-modifying. Therapeutic approaches include lifestyle changes, the use of hormonal contraceptives and off-label use of agents known to affect the reproductive endocrine and/or metabolic aspects of the disease (e.g., metformin).

PCOS is associated with an elevated risk of cardiovascular disease, type 2 diabetes mellitus, metabolic dysfunction associated steatotic liver disease, mental health disorders [e.g., major depressive disorder (MDD) and anxiety disorder] and overall decreased health-related quality of life [10, 37]. Extant literature reports that persons with PCOS are 2.5 times more likely to be diagnosed with MDD, based on Diagnostic and Statistical Manual of Mental Disorders (DSM) criteria, compared to non-PCOS populations, with depressive symptoms remaining consistently high across their lifespan [4, 10, 14]. In addition, the rate of PCOS is higher in women with bipolar disorder after accounting for the iatrogenic contribution of valproate [21, 22, 45]. Women with PCOS were also found to be at increased risk of developing eating disorders, personality disorders, tics, schizophrenia, and autism spectrum disorders [4, 6].

Suicide is a leading cause of death that is multi-faceted and can include domains such as suicidal ideation, suicidal behaviours (e.g., suicide attempts), and completed suicide [9, 15, 16, 27, 32, 44]. Extant literature indicates that persons with chronic medical conditions are also differentially at an elevated risk for death by suicide compared to the general population [25, 28]. In light of the replicated association between PCOS and elevated rates of depressive disorders, it is crucial to explore how these mental health challenges may further contribute to the risk of suicide in this underserved population. Specifically, the pathophysiology of PCOS (i.e., insulin resistance, metabolic syndrome, disrupted neuroendocrine function) substantially overlap with that of depressive disorders, which may further exacerbate suicide risk [24]. In addition to biological factors, psychosocial factors such as decreased health-related quality of life and elevated chronic stress, which are commonly observed in persons with PCOS, may further contribute to suicide risk. Moreover, hopelessness is a commonly reported symptom in both persons with PCOS and MDD, which has been implicated as a significant predictor of suicidal ideation and attempts [3, 30, 34]. Therefore, overlapping biological and psychosocial risk factors between PCOS and depression may also contribute to elevated suicide risk. Taken together, the foregoing observations instantiate a need to evaluate the risk of suicidality in persons with PCOS. Herein, we sought to determine whether an association between PCOS and suicidality exists. Secondarily, we aimed to disaggregate aspects of suicidality (i.e., suicidal ideation, suicidal behaviours, completed suicide) and synthesize a summary of current literature reporting on the relative risk in the PCOS population compared to non-PCOS populations.

## Methods

### Search strategy and databases

This systematic review was conducted in accordance with the 2020 Preferred Reporting Items for Systematic Reviews and Meta-Analyses (PRISMA) guidelines [31]. To investigate the association of PCOS with aspects of suicidality, a systematic search was conducted on PubMed, Ovid (i.e. Medline, Embase, APA PsychInfo, AMED), and Scopus databases from inception to January 7, 2025. The aforementioned databases were searched using the following search string: ((Polycystic ovarian syndrome) OR (PCOS) OR (Polycystic ovary syndrome) OR (Hyperandrogenic anovulation) OR (Stein-Leventhal syndrome)) AND ((Suicide*) OR (Suicidality) OR (Suicidal ideation) OR (Suicidal behavi*) OR (Suicide attempt*) OR (Completed Suicide)). To ensure all relevant articles were retrieved, a manual search was conducted through citation searching and on Google Scholar.

### Eligibility criteria

Studies were eligible for inclusion if they met the following inclusion criteria: (1) Participants must have a confirmed diagnosis of PCOS, (2) Study must report on the effects of and/or the association between a PCOS diagnosis and a suicide outcome (i.e., suicidality composite scores, suicidal ideation, suicidal behavior including suicide attempts, completed suicide), (3) participants must be within reproductive age (i.e., 15–49 years), (4) must be primary research (e.g., case–control studies, cohort studies, cross-sectional studies. Studies were excluded if they met at least one of the following exclusion criteria: (1) non-human studies (i.e., in vitro, animal studies), (2) participants do not have a confirmed diagnosis of PCOS, (3) study does not report on suicide outcomes, (4) non-primary research (e.g., reviews, commentaries, etc.), (5) Case studies or case series, (6) Study is not published in English. The eligibility criteria are displayed in Table 1.

**Table 1:** Eligibility Criteria

Inclusion	Exclusion
● Participants must have a confirmed diagnosis of PCOS ● Study must evaluate the effects of and/or the association between PCOS diagnosis and suicide outcomes (i.e., suicidality, suicidal ideation, suicidal behaviour, suicide attempts, completed suicide) ● Participants must be within reproductive age (i.e., 15–49 years) ● Primary research (e.g., case–control studies, cohort studies, cross-sectional studies)	● Non-human studies (i.e., in vitro, animal studies) ● No confirmed diagnosis of PCOS ● Does not report on suicide outcomes ● Non-primary research (e.g., reviews, commentaries, etc.) ● Case studies or case series ● Not published in English

### Study screening

Two reviewers (S.W. and G.H.L.) independently screened all retrieved studies using the Covidence platform [7]. The retrieved studies were first screened for relevance by title and abstract. Any studies that received at least one vote of relevance were then full-text screened against the eligibility criteria (Table 1). To be included in this review, studies must have received a unanimous decision for inclusion by both reviewers. Any discrepancies were resolved through discussion.

### Data extraction

The studies that were included were extracted for relevant data using a piloted data extraction table. Data to be extracted were established a priori and included: (1) author and date of publication, (2) study design, (3) sample size, (4) sample age, (5) the suicide domain(s) evaluated, (6) results reporting on suicidality (i.e., suicidal ideation, suicidal behaviour, completed suicide) within the sample or the association between PCOS and domains of suicidality.

### Methodological quality and risk of bias assessment

The included studies were assessed for their methodological quality and potential risk of bias using the National Institute of Health (NIH) risk of bias tools based on the study design of the component study [26]. Case–control studies were evaluated using the NIH quality assessment tool of case–control study tool [26]. Cohort and cross-sectional studies were evaluated using the NIH quality assessment tool of observational cohort and cross-sectional studies [26]. Studies were assessed independently by two reviewers (S.W. and G.H.L.). Any discrepancies were resolved through discussion.

## Results

### Search results

**Fig. 1:**
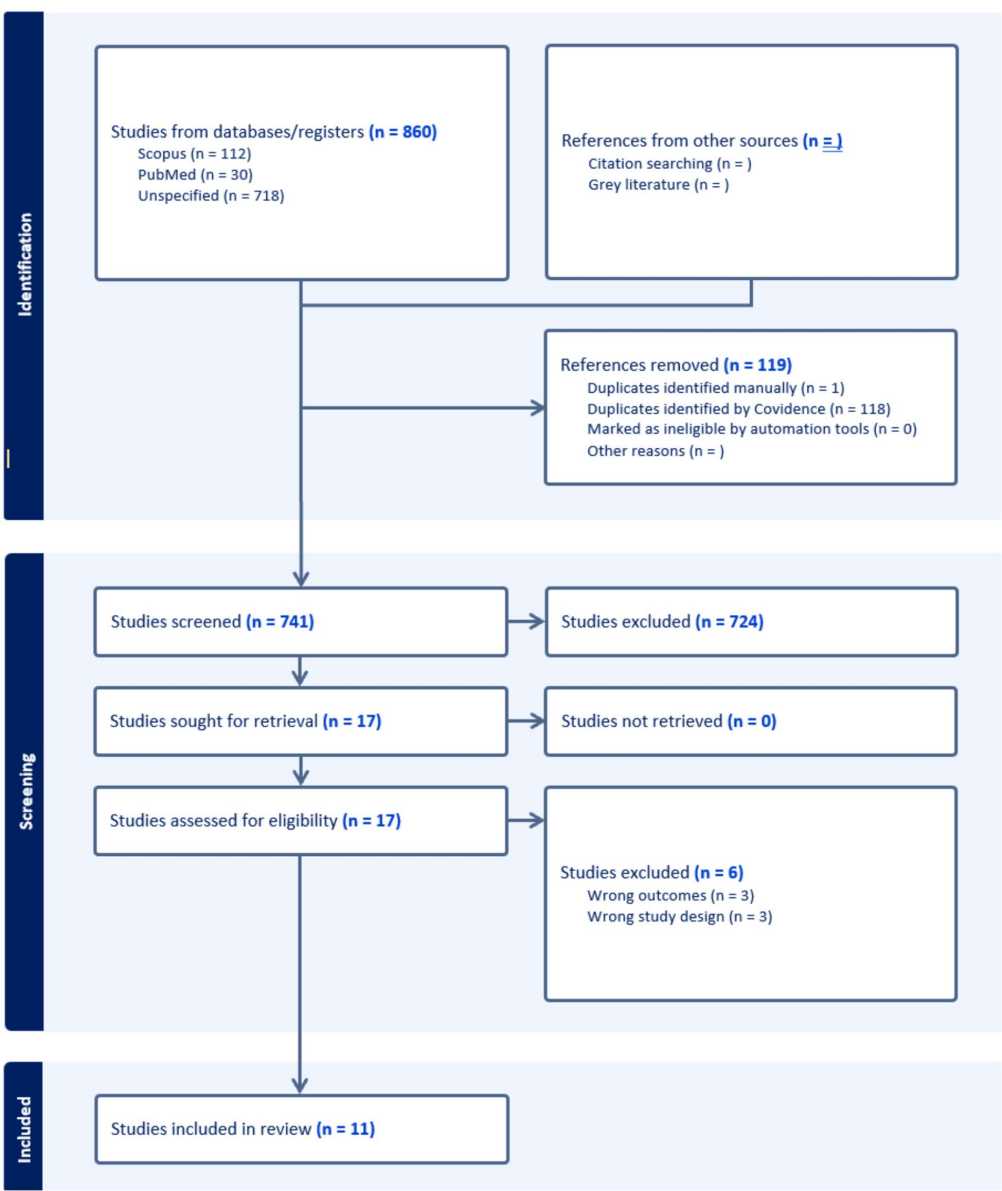
Study Screening Process. Study screening and figure generation was conducted on Covidence

The search resulted in the identification of 860 articles. Following the removal of duplicates, 741 studies underwent title and abstract screening, of which 17 studies were deemed relevant. Of the 17 studies, 11 studies were eligible to be included in this review (n = 11) (Table 2). Studies were excluded due to not reporting suicide outcomes (n = 3) or due to incorrect study design (i.e., editorials, qualitative studies) (n = 3). The component studies consist of 2 case–control studies, 3 cohort studies, 4 cross-sectional studies, and 2 chart review studies. See Fig. 1 for further information on the study screening process.

**Table 2:** Characteristics and Main Results of Included Studies

Study	Study Design	Sample Size	Age	Suicide Domain	Psychiatric Diagnoses	Suicidality Results
Almis et al. [2]	Case–control	314 total participants 153 participants with PCOS 161 healthy controls	PCOS mean: 15.57 (1.11) Control mean: 15.71 (1.05)	Presence of current suicidal ideation	Not reported	Prevalence rates of suicidal ideation were significantly greater in the PCOS group (17 (11.1%)) compared to the healthy controls (9 (5.6%))
Cesta et al. [6]	Retrospective cohort study	268 235 total participants 24 385 participants with PCOS 243 850 healthy controls	Not reported	History of suicide attempt and completed suicide	Psychiatric diagnoses were significantly greater in PCOS participants compared to controls (OR = 1.56, 95% CI = [1.51, 1.61]) Bipolar Disorder: OR = 1.91, 95% CI = [1.73, 2.10]); AOR = 1.41, 95% CI = [1.28, 1.56]) Depressive disorders, any: OR = 1.56, 95% CI = [1.50, 1.63]), AOR = 1.25, 95% CI = [1.19, 1.31]) Severe depression: OR = 1.45, 95% CI = [1.32, 1.60]), AOR = 1.06, 95% CI = [0.96, 1.18])	Persons with PCOS had a significantly greater odds of attempted suicide (OR = 1.41, 95% CI = [1.31, 1.52]), but was not significant upon adjustment (AOR = 1.05, 95% CI = [0.98, 1.14]) Completed suicides were nonsignificantly different between the PCOS and control group (OR = 1.19, 95% CI = [0.71, 2.02]; AOR = 0.86, 95% CI = [0.51, 1.46])
Gomaa et al. [11]	Cross-sectional study	60 total participants 30 participants with PCOS and infertility 30 participants with non-PCOS infertility	PCOS mean: 27.2 (6.2) non-PCOS mean: 29.2 (5.7)	Lifetime history of suicidal ideation and attempts	Diagnosis (PCOS vs controls): MDD: 53.33% vs 23.33% Other diagnoses: 13.33% vs 13.33% No diagnosis: 33.33% vs 63.33%	Lifetime history of suicidal ideation was not significantly different between the PCOS (13, 43.4%) and non-PCOS (11, 36.7%) participants (p = 0.60) Lifetime history of suicide attempts were also not significantly different between PCOS (2, 6.7%) and non-PCOS (2, 6.7%) participants (p = 1.00)
Hussain et al. [13]	Cross-sectional study	150 total participants 110 participants with PCOS 40 healthy controls	PCOS mean: 24.77 Controls: 22.65	History of suicidality	Psychiatric comorbidity was significantly greater in the PCOS group compared to the control group (52.7% vs 10%, respectively) MDD was significantly greater in PCOS compared to controls (23.64% vs 7.5%, respectively). Consistent with BPAD, dysthymia, GAD, agoraphobia, OCD, panic disorder and PTSD	Suicidality was observed in 9 (8.18%) PCOS participants. None of the healthy controls reported suicidality
Hsu et al. [12]	Prospective cohort study	208 560 total participants 18 960 participants with PCOS 189 600 healthy controls	PCOS mean: 27.71 (7.00) Controls mean: 27.71 (7.02)	Suicide attempts	Prevalence of all psychiatric disorders (i.e., Schizophrenia, Bipolar Disorder, Depressive Disorder, Alcohol Use Disorder, Substance Use Disorder) were equivalent between groups	PCOS patients had a higher prevalence of suicide attempts compared to the controls (SMD = 1.23). Risk for suicide was 8.47 times greater in the PCOS group compared to the healthy controls (Hazard ratio = 8.47, 95% CI = [7.54, 9.51]), which was observed over 16 years of follow-up Suicide attempts occurred at an earlier age compared to controls (SMD = 0.38) and a shorter time between enrollment and suicide attempt occurrence (SMD = 0.64)
Månsson et al. [19]	Case–control study	98 total participants 49 participants with PCOS 49 healthy controls	Total mean: 35.9 (10.4)	History of suicide attempts	MDE, social phobia, and eating disorders were significantly greater in the PCOS group compared to the control group Any major depressive episode: OR = 3.8 95% CI = [1.5, 8.7] Recurrent depressive episodes: OR = 3.8, 95% CI = [1.5, 9.5] Manic/hypomanic episodes, panic disorders, GAD, OCD, and bulimia nervosa were not significantly different	Suicide attempt status was significantly greater in the PCOS group compared healthy controls (OR = 8.3, 95% CI = [1.0, 70])
Maya et al. [20]	Chart review	493 total participants 447 overweight and obese adolescents 46 overweight and obese adolescents with PCOS	Total sample, median (range): 17 (14–22)	History of self-harm and/or suicidality	Depression and/or anxiety was observed in 17 (37.0%) PCOS patients and in 129 (33.0%) non-PCOS patients (p = 0.59) indicating no significant difference in observed psychiatric diagnoses	Rates of self-harm and/or suicidality in the PCOS patients were 8 (17.4%) and 67 (17.1%) in the non-PCOS population (p = 0.97) There was no significant difference in self-harm and/or suicidality in PCOS patients compared to non-PCOS patients (OR = 0.86, 95% CI = [0.32, 2.31]), p = 0.76)
Scaruffi et al. [35]	Cross-sectional study	94 total participants 49 participants with PCOS 45 healthy controls	PCOS mean: 25.8 (4.7) Control mean: 25.2 (5.9)	Suicidality: Rorschach indices (suicide constellation; S-Con)	PCOS group had a significantly greater rate of mental disorders compared to the control group	The presence of suicidality was significantly greater in the PCOS group compared to the control group (8.1% and 6%, respectively, p < 0.05). Presence of personality and psychiatric disorders were higher in this population
Schweisberger et al. [36]	Retrospective chart review	390 total patients 157 patients with PCOS 233 patients without PCOS	PCOS mean: 16.16 (1.58) No PCOS (site 1) mean: 15.89 (1.47) No PCOS (site 2) mean: 15.78 (1.46)	History of suicidality	Rates of depression were significantly greater in the transgender group (100%) compared to the cisgender group (37.6%) (p < 0.01) which was similarly observed with anxiety (77.% vs 35.8%, respectively, p = 0.03)	Rates of suicide history in cisgender PCOS patients: 18/125 (14.4%) Rates of suicide history in transgender PCOS patients: 4/11 (36.4%) Trend of increased prevalence of suicide in transgender PCOS patients compared to cisgender (p = 0.08)
Trivedi et al. [39]	Cohort study	11 985 total participants 3995 participants with PCOS 7990 healthy controls	PCOS mean: 16.03 (0.03) Control mean: 16.03 (0.03)	Suicidal ideation/attempts	Mood disorders were significantly greater in the PCOS group compared to the control group (43.6% vs 33.1%, OR = 1.56, p < 0.01). Within the PCOS group the rates for MDD was 36.5% and bipolar disorder were 5.5% Anxiety was significantly higher in the PCOS group compared to the control group (37.5% vs 25.1%, OR = 1.80, p < 0.001) No significant differences in adjustment disorders and schizophrenia	Suicidal ideation/attempt was nonsignificantly different between the PCOS and control group (19.6% and 18.3%, respectively, p = 0.74) Study did not aggregate suicidal ideation and attempts
Williams et al. [41]	Cross-sectional study	418 total participants 113 participants with PCOS 305 healthy controls	PCOS mean: 31.01 (6.33) Controls mean: 33.65 (11.38)	Recent suicidal ideation, non-suicidal self-injury, future suicidal intention	Not reported	Recent suicidal ideation was significantly greater in the PCOS participants compared to the healthy controls (t = −4.21, p < 0.001, 95% CI = [−0.730, −0.266]), d = 0.45) Non-suicidal self-injury was significantly greater in the PCOS group (t = −2.04, p = 0.04, 95% CI = [−0.746, −0.013], d = 0.22) Similarly, future suicidal intention was significantly greater in the PCOS group compared to the control group (t = −2.33, p = 0.02, 95% CI = [−0.598, −0.051], d = 0.25) PCOS was positively correlated with greater suicidal ideation (r = 0.202, p < 0.001), non-suicidal self-injury (r = 0.099, p < 0.05) and suicidal intention (r = 0.113, p < 0.05)

*CI* confidence interval, *GAD* generalized anxiety disorder, *HR* hazard ratio, *MDD* major depressive disorder, *MDE* major depressive episode, *OCD* obsessive–compulsive disorder, *OR* odds ratio, *PCOS* polycystic ovarian syndrome, *PTSD* posttraumatic stress disorder, *SMD* standardized mean difference

### Risk of bias results

When considering the case–control studies, both studies received a quality rating of “good” and were deemed to have a low risk of bias; however, one area of concern was not reporting a sample size justification (Supplementary Table 1) [2, 17]. From the cohort and cross-sectional studies, four studies received a quality rating of “good” while the other three were “fair” (Supplementary Table 2). Some concerns were similar to the case–control studies wherein sample size justifications, power descriptions and effect size estimates were not provided [6, 11, 13, 35, 39]. An additional domain that may introduce a potential bias is inadequate reporting of the blinding integrity of the assessors, which was present across all of the cohort and cross-sectional studies. Two included studies are chart reviews, which could not be evaluated for their risk of bias as there is no validated and established risk of bias tool designed to assess chart review studies [20, 36]. However, an area that may introduce potential bias is adequately reporting sample selection and inclusion criteria as well as choosing an adequate time frame to observe outcomes of interest.

### Association of PCOS with suicidality composite scores

We identified five studies (n = 5) that reported on the association between PCOS and suicidality, but did not disaggregate between the different domains of suicidality investigated (Table 2) [13, 20, 29, 36]. Across the 13,112 participants within the identified studies, mixed results are reporting on suicidality in persons with PCOS. Specifically, Hussain et al. [13] and Scaruffi et al. [35] observed that persons with PCOS had higher rates of suicidal ideation and/or behaviour compared to the control group (8.0–8.18% vs 0–6.0%, respectively),however, the studies did not report on the strength of association of these results.

In contrast, Maya et al. [20] reported suicidality as a composite score of self-harm and/or suicidal ideation history wherein there were nonsignificant differences between persons with PCOS (n = 46) and the control group (n = 447) were observed (OR = 0.86, 95% CI = [0.32, 2.31]); a trend similarly observed by Trivedi et al. [39] (19.6% vs 18.3%, respectively, p = 0.74). Furthermore, when analyzed for the effects of gender on composite scores of suicidal ideation and/or behavior history in persons with PCOS, there were also nonsignificant differences between cisgender and transgender individuals (14.4% vs 36.4%, p = 0.08) [36].

Notwithstanding the mixed results, the presence of at least one psychiatric comorbidity was more commonly observed in persons with PCOS compared to the control group [13, 20, 35, 39]. In general, the most commonly reported psychiatric disorders within the PCOS group were MDD, bipolar disorder and any anxiety disorder (Table 2). As psychiatric disorders are strongly linked to suicide risk, further investigation is required to evaluate the strength of association between PCOS and suicide risk after adjustment for a psychiatric diagnosis.

#### Association of PCOS with suicidal ideation

Three out of eleven of the included studies reported on the prevalence of suicidal ideation in persons with PCOS (Table 2) [2, 11, 41]. The association of PCOS with suicidal ideation was investigated across a total of 792 participants. Notably, Gomaa et al. [11] reported that across 60 participants, there were nonsignificant differences in lifetime history of suicidal ideation between persons with PCOS (43.4%) and non-PCOS (36.7%) participants (p = 0.60). Notwithstanding the nonsignificant difference in suicidal ideation history, the PCOS group had a higher prevalence of MDD diagnoses compared to the control group [11]. Contrastingly, in terms of recent suicidal ideation status, Williams et al. [41] indicate that participants with PCOS were more likely to have recent suicidal ideation compared to the control group (n = 418, t = − 4.21, p < 0.001, 95% CI = [− 0.730, − 0.266], d = 0.45). This was also replicated for current suicidal ideation status when comparing persons with PCOS and non-PCOS participants (11.1% vs 5.6%, respectively) [2]. The studies conducted by Williams et al. [41] and Almis et al. [2] did not measure psychiatric comorbidities in their samples.

#### Association of PCOS with suicidal behaviour

##### Self-injury

From the included studies, Williams et al. [41] reported on the prevalence of suicidal behavior, specifically non-suicidal self-injury (NSSI), in persons with PCOS compared to non-PCOS individuals (n = 418). While this study did not report on the prevalence of psychiatric diagnoses in their sample, NSSI was significantly more prevalent in the PCOS group compared to the non-PCOS group (t = − 2.04, p = 0.04, 95% CI = [− 0.746, − 0.013], d = 0.22]) [41]. Similar trends were also observed for future suicidal intention (t = − 2.33, p = 0.02, 95% CI = [− 0.598, − 0.051], d = 0.25) [41]. The foregoing result instantiates the need to investigate the prevalence of suicidal behaviour (e.g., self-injury, preparatory acts of suicide attempts, suicide planning, etc.) in persons with PCOS as well as the prevalence of psychiatric comorbidities in these individuals. Therefore, additional large, adequately-controlled clinical trials are required to further evaluate the prevalence and risk of self-injurious behaviours in PCOS populations.

##### Suicide attempts

Four of the eleven included studies reported on the prevalence and/or the risk of suicide attempts in persons with PCOS compared to controls (n = 476,953) (Table 2) [6, 11, 12, 19]. Individuals with PCOS (n = 49) were reported to have significantly greater odds of suicide attempt history compared to the control group (n = 49) (Odds ratio; OR = 8.3, 95% CI = [1.0, 70]), which may be attributable to the higher prevalence of any major depressive episode (OR = 3.8, 95% CI = [1.5, 8.7]) or recurrent major depressive episodes (OR = 3.8, 95% CI = [1.5, 9.5]) [19]. However, while individuals with PCOS were more likely to have attempted suicide in their lifetime (OR = 1.41, 95% CI = [1.31, 1.52]), Cesta et al. [6] in a retrospective cohort study of 268,235 participants reported that the association was no longer significant after adjusting for psychiatric diagnoses (adjusted OR = 1.05, 95% CI = [0.98, 1.14]). The foregoing result was similarly observed by Gomaa et al. (2023) wherein there was a higher prevalence of MDD in the PCOS group compared to the non-PCOS group; however, no significant difference in suicide attempt history between PCOS and non-PCOS participants (both 6.7%) were observed.

In contrast, in a 16-year follow-up study using a national database (n = 208, 56), Hsu et al. (2024) reported that individuals with PCOS had a higher prevalence of suicide attempts compared to controls (standardized mean difference; SMD = 1.23) and are at a higher risk of future suicide risk (hazard ratio; HR = 8.47, 95% CI = [7.54, 9.51]). Moreover, suicide attempts were found to occur more prominently at earlier ages in individuals with PCOS compared to the controls (SMD = 0.38) wherein older adults with PCOS had the lowest risk of suicide (HR = 3.75, 95% CI = 2.23, 6.28]) compared to adolescents (HR = 5.38, 95% CI = [3.93, 7.37]) and young adults (HR = 9.15, 95% CI = [8.03, 10.42]) [12]. The aforementioned trends were suggested not to be subserved by a psychiatric comorbidity as there was an equivalent prevalence of psychiatric disorders between groups [12].

The aforementioned results suggest that individuals with PCOS are at an increased risk of suicide attempts; however, the underlying pathophysiological and psychopathological mechanisms require further investigation. Moreover, whether the risk of suicide is direct and independent of comorbid mental illness and/or whether causality can be attributed to both PCOS and mental disorders is not sufficiently addressed by the studies we have identified.

#### Association of PCOS with completed suicide

We identified one study that evaluated the prevalence of completed suicide cases in persons with PCOS (n = 268,235) [6]. Based on the retrospective cohort data, completed suicides were nonsignificantly different between the PCOS cohort and the control cohort (OR = 1.19, 95% CI = [0.71, 2.02]; adjusted OR = 0.86, 95% CI = [0.51, 1.46]). Notably, the prevalence of a mental health disorder was significantly greater in the PCOS population compared to the control cohort (OR = 1.56, 95% CI = [1.51, 1.61]). The aforementioned result suggests that the presence of a mental health disorder is a greater contributor to completed suicide risk compared to PCOS. However, the degree to which PCOS may contribute to completed suicide risk requires further investigation.

## Discussion

Our results indicate that individuals with PCOS may be at an increased risk for multiple aspects of suicidality (i.e., suicidal ideation, suicidal behaviour, suicide attempts) compared to non-PCOS populations. Specifically, persons with PCOS were more likely to endorse current suicidal ideation, but not a history of suicidal ideation. In addition, this was also consistent with suicide attempts wherein persons with PCOS were more likely to endorse a lifetime history of suicide attempts and were at an increased risk of future suicide attempts compared to the control groups. In terms of suicidal behaviour, while one study reported that persons with PCOS are at a greater odds of engaging in nonsuicidal self-injury compared to the control groups, there was a lack of studies reporting on the presence of other suicidal behaviours, which warrants further investigation. This notion is also seen with completed suicide cases as we identified one study that reported nonsignificant odds of completed suicide cases in persons with PCOS.

Notwithstanding the increased prevalence and risk of suicide in PCOS populations, the presence of a comorbid psychiatric disorder may conflate the rates and severity of measured suicide outcomes. Consensus exists that persons with PCOS are at an increased risk of psychiatric comorbidities [14, 21]. A highly replicated finding is that PCOS differentially affects persons with MDD and bipolar disorder [5]. However, while some studies reported an increased prevalence of psychiatric comorbidities in their PCOS sample, suicidality rates were not consistently elevated [6, 11, 19]. Whether the measured suicide outcomes are a result from PCOS and/or psychiatric disorder pathophysiology has yet to be determined.

Notably, both PCOS and depressive disorders have overlapping pathophysiology that may contribute to suicide development and prognosis. For example, both persons with PCOS and depressive disorders are commonly reported to evince metabolic disruption, insulin resistance and alterations in the hypothalamic–pituitary–adrenal axis [15]. In particular, insulin resistance is a hallmark in PCOS, similar to those observed in type 2 diabetes mellitus [29]. This metabolic dysfunction affects various biological pathways also implicated in depressive disorders. Insulin resistance, in particular, impacts neurotransmitter systems such as dopamine, which plays a crucial role in the phenomenology and treatment of depressive disorders [23]. Dysregulation in dopamine signaling, often due to brain insulin resistance, affects reward processing and can contribute to depressive behaviors and suicide risk [8]. The overlapping mechanisms between PCOS, suicidality, and insulin resistance underscore the importance of considering insulin modulation as a potential therapeutic target.

Antidiabetic drugs that improve insulin sensitivity might not only manage metabolic symptoms in PCOS and type 2 diabetes mellitus but may also alleviate depressive symptoms and reduce suicide risk [1, 33, 40]. Extant literature commonly reports insulin resistance to be a commonly reported symptom in both PCOS and depressive disorders as well as a key factor implicated in the disease pathology of both disorders [15]. Therefore, the underlying biological mechanisms that subserve both PCOS and depressive disorders may exacerbate domains of psychopathology (e.g., suicidal ideation and behaviour, reward impairment, cognitive dysfunction) that can lead to decreased treatment efficacy, worsening disease prognosis and an overall decreased health-related quality of life and functional capacity [17, 18, 42].

This systematic review presented herein has methodological limitations that may limit our inferences and interpretation of the results. Primarily, there are inconsistencies in the tools used to measure the presence and severity of suicidality in the study samples. In addition, suicidality is polysemous and differences in the suicide domain being evaluated may affect how we measure the association between diagnoses and suicidality. As psychiatric comorbidity was not consistently measured and/or was not adjusted for in suicide analyses, we cannot fully ascertain whether the presence of suicidality is associated with PCOS or a comorbid psychiatric disorder and whether there is discrete and/or overlapping pathophysiology. Specifically in terms of bipolar disorder, we are unable to evaluate differences in the frequency of manic, mixed, and depressive episodes in persons with PCOS compared to non-PCOS populations, which may further contribute to suicide risk. Due to a dearth of studies evaluating and distinguishing between the suicidality domains (i.e., suicidal ideation, suicidal behaviour, completed suicide) as well as differences in sample ages and overall study design, we were not able to conduct a meta-analysis or a quantitative analysis of the literature. Consequently, we are unable to evaluate how suicidality changes over time across the PCOS disease prognosis.

## Conclusion

Our results indicate that PCOS is associated with an increased risk of suicide and associated psychiatric comorbidities. Therefore, persons with PCOS should be routinely evaluated for the presence of clinically significant depressive symptoms as well as aspects of suicidality. Whether increased suicidality in PCOS populations is a direct effect of the disease state and/or is largely moderated by psychiatric comorbidity is a vista for future research.

## Supplementary Information

Supplementary Material 1.

## Data Availability

No datasets were generated or analysed during the current study.

## Abbreviations

HR: Hazard ratio
PCOS: Polycystic ovarian syndrome
NSSI: Non-suicidal Self-Injury
OR: Odds ratio
SMD: Standardized mean difference
