# Supplementary material for: Suicide risk in persons with polycystic ovarian syndrome: a systematic review
Source: Ann Gen Psychiatry. 2025 Jun 2;24:38. doi: 10.1186/s12991-025-00574-w (PMC12128235; doi:10.1186/s12991-025-00574-w)
Supplement: Supplementary file 1 — Supplementary Material 1. [file 12991_2025_574_MOESM1_ESM.docx]

**Supplementary Material**

**Table 1.** Case-Control Study Risk of Bias Assessment

| **Study** | **Item** | | | | | | | | | | | | **Quality Rating** |
| --- | --- | --- | --- | --- | --- | --- | --- | --- | --- | --- | --- | --- | --- |
|  | **1** | **2** | **3** | **4** | **5** | **6** | **7** | **8** | **9** | **10** | **11** | **12** |  |
| Almis et al. (2021) | Yes | Yes | No | Yes | Yes | Yes | CD | Yes | Yes | Yes | NR | Yes | **Good** |
| Månsson et al. (2008) | Yes | Yes | No | Yes | Yes | Yes | Yes | Yes | Yes | Yes | No | Yes | **Good** |

* Studies were assessed using the NIH quality assessment tool of case-control study tool (NIH, 2013).

**Table 2.** Cohort and Cross-sectional Study Risk of Bias Assessment

| **Study** | **Item** | | | | | | | | | | | | | | **Quality Rating** |
| --- | --- | --- | --- | --- | --- | --- | --- | --- | --- | --- | --- | --- | --- | --- | --- |
|  | **1** | **2** | **3** | **4** | **5** | **6** | **7** | **8** | **9** | **10** | **11** | **12** | **13** | **14** |  |
| Cesta et al. (2016) | Yes | Yes | CD | Yes | No | Yes | Yes | NA | Yes | NA | CD | No | NA | Yes | **Fair** |
| Gomaa et al. (2023) | Yes | Yes | Yes | Yes | No | Yes | Yes | NA | Yes | NA | Yes | No | Yes | Yes | **Good** |
| Hussain et al. (2015) | Yes | Yes | Yes | Yes | No | Yes | Yes | NA | Yes | NA | Yes | No | Yes | Yes | **Good** |
| Hsu et al. (2024) | Yes | Yes | CD | Yes | Yes | Yes | Yes | NA | Yes | NA | Yes | No | NA | Yes | **Good** |
| Scaruffi et al. (2014) | Yes | Yes | Yes | Yes | No | Yes | Yes | NA | Yes | NA | Yes | No | Yes | No | **Fair** |
| Trivedi et al. (2024) | Yes | Yes | CD | Yes | No | Yes | Yes | NA | Yes | NA | Yes | No | NA | No | **Fair** |
| Williams et al. (2022) | Yes | Yes | Yes | Yes | Yes | Yes | Yes | NA | Yes | NA | Yes | No | Yes | Yes | **Good** |

* Studies were assessed using the NIH quality assessment tool of observational cohort and cross-sectional studies (NIH, 2013).
